# Post-Myocardial Revascularization: As a Nidus for an Electrical Storm!

**DOI:** 10.7759/cureus.43450

**Published:** 2023-08-14

**Authors:** Najlaa Belharty, Tanae El Ghali, Selma Siagh, Zakaria Choho, Fatima Azzahra Benmessaoud, Ibtissam Fellat, Latifa Oukerraj, Mohamed Cherti

**Affiliations:** 1 Department of Cardiology B, Ibn Sina Hospital, Mohammed V University, Rabat, MAR; 2 Department of Cardiology B, Ibn Sina Hospital, Mohammed V university, Rabat, MAR

**Keywords:** ventricular arrhythmias, ventricular tachycardia, myocardial revascularization, reperfusion injury, electrical storm

## Abstract

Electrical storm (ES) is a critical and potentially life-threatening cardiac rhythm disorder. It is characterized by the presence of three or more distinct episodes of sustained ventricular tachycardia (VT) or ventricular fibrillation (VF) that necessitate appropriate termination. ES may occur in the setting of acute myocardial infarction or following myocardial reperfusion. An urgent treatment approach is necessary for better outcomes. We represent a case of a 64-year-old patient who presented with sudden chest pain and an episode of palpitations related to non-ST elevation myocardial infarction (NSTEMI), who has undergone percutaneous coronary intervention of the stenotic epicardial artery, but subsequently experienced an ES in the absence of stent thrombosis. ES presented in the form of sustained monomorphic VT that required synchronous direct current cardioversion, anti-arrhythmic drugs, deep sedation, and endotracheal intubation with a favorable course, with the patient being discharged after 14 days hospital stay. The practitioner should be mindful of the potential occurrence of ES following myocardial revascularization and should tailor the management approach.

## Introduction

Electrical storm (ES) refers to the occurrence, in a 24-hour window, of malignant ventricular arrythmias (VAs), three or more prolonged episodes of ventricular fibrillation (VF) or a hemodynamically unstable ventricular tachycardia (VT), or, in a 12 hours’ span, a persistent VA in spite of attempts of termination [1,2]. ES carries an adverse prognosis; the majority of patients have a high mortality rate, with many succumbing within a short period, often minutes or hours, particularly if they have a recent history of myocardial infarction (MI) or persistent myocardial ischemia [3], and approximately 6% of cases involving acute coronary syndrome (ACS) are complicated by VA. The presence of irreversible myocardial ischemia leads to the development of focal and non-focal arrhythmogenic lesions that may progress into VF or VT [4]. Interestingly, as we will demonstrate in our case, arrhythmias that arise during the period of ischemia/reperfusion could be attributed to ischemia, no-reflow phenomenon following the opening of the infarct-related coronary artery, or reperfusion injury [5].

## Case presentation

**Figure 1:**
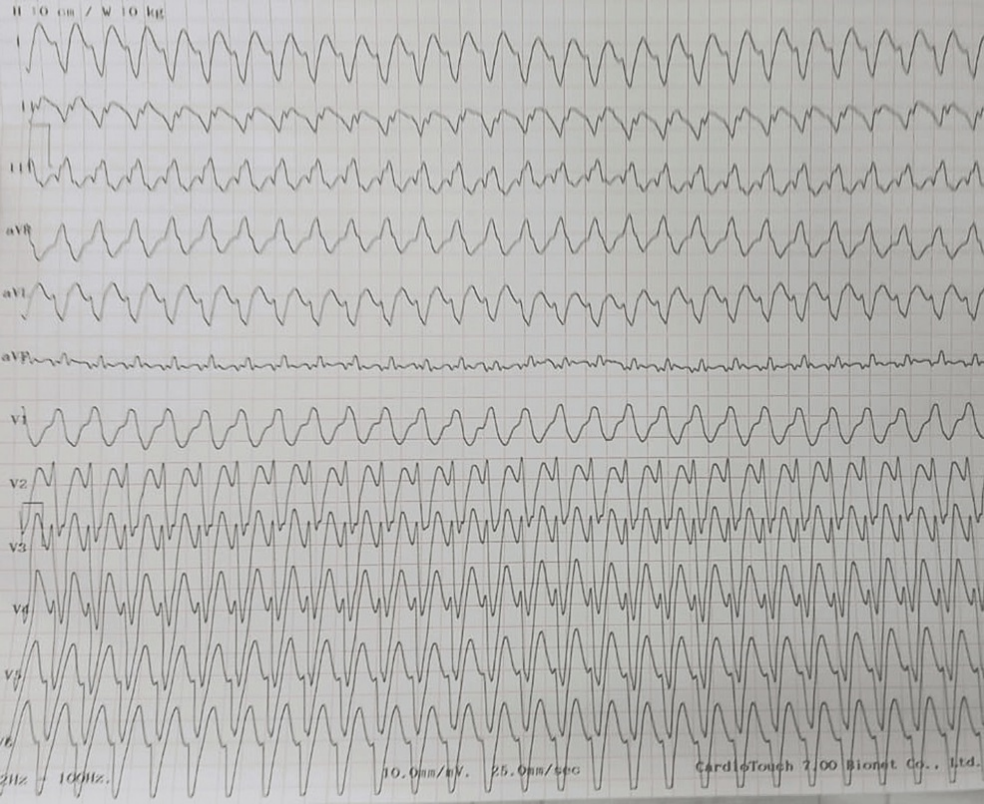
Electrocardiogram showing monomorphic ventricular tachycardia with a ventricular rate of 180 beats per minute

A 64-year-old patient with cardiovascular risk factors, including pre-diabetes, dyslipidemia treated with statins, and second-hand smoke exposure, and a medical history of thyroidectomy with hormone replacement therapy five years ago, as well as a history of chronic obstructive pulmonary disease treated with beta-2 agonists, was admitted to our facility, complaining of a sudden, severe chest pain, radiating to his arms associated with diaphoresis and palpitations. His medical history also includes the occurrence of infero-lateral MI a year before, which was related to a severe lesion of the obtuse marginal artery, whose presumed territory was considered non-viable on stress echocardiography. Initial vital parameters were as follows: pulse of 180 beats/min, respiration rate of 22 cycles/min, blood pressure of 80/66 mmHg, and oxygen saturation of 97%. Electrocardiogram (ECG) showed a sustained VT at a rate of 180 beats/min. Being hemodynamically compromised, the patient received synchronized direct current (DC) cardioversion with restoration of sinus rhythm (Figure 1).

**Figure 2:**
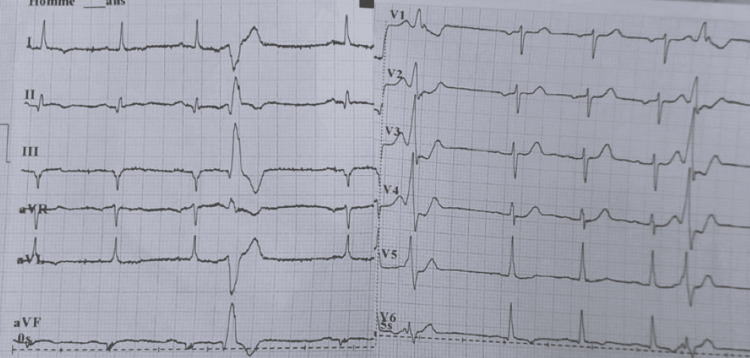
Electrocardiogrampost-electric cardioversion showing sinus rhythm at a rate of 75 beats per minute, with isolated monomorphic ventricular ectopic beats

Baseline ECG showed negative infero-lateral T waves along with Q waves in the same territory, and a frontal microvoltage (Figure 2).

The patient received a loading dose of aspirin and clopidogrel and was urgently transferred to the catheterization lab for coronary assessment.

**Figure 3:**
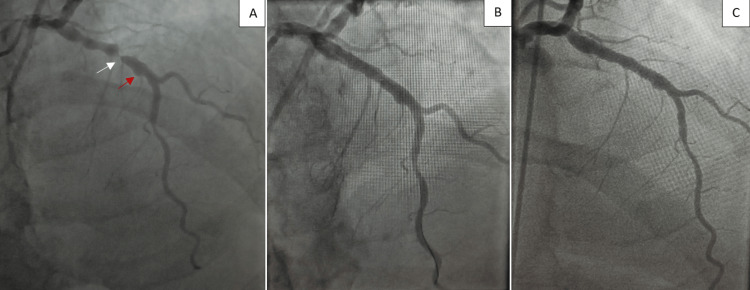
A. Coronary angiography demonstrating staged lesions of proximal LAD artery, with a distal 90% stenosis (white arrow), followed by an aneurysmal portion (red arrow) and TIMI flow grade 2. B. Successful angioplasty of the LAD. C. Control coronary angiography showing no stent thrombosis and TIMI flow grade 3. LAD, left anterior descending; TIMI, thrombolysis in myocardial infarction

Coronary angiography (CAG) revealed staged coronary artery lesions of the proximal left anterior descending artery (LAD), with the distal lesion with an estimated 90% stenosis, followed by a coronary aneurysm, with a distal thrombolysis in myocardial infarction (TIMI) flow grade 2. We proceeded by stenting the lesion, sparing the aneurysmal portion, and yielding an optimal result (Figures 3A, 3B). A few ventricular ectopic beats were recorded on ECG monitoring.

The patient was then retransferred to the cardiac ward. After an hour, the patient experienced recurrent episodes of sustained monomorphic VT that required synchronous cardioversion by a biphasic 200-J DC shock and administration of amiodarone. We ran laboratory testing that showed no electrolyte imbalances that could have triggered ES.

After restoration of hemodynamic stability, control CAG was performed, showing an appropriately positioned stent with no thrombi (Figure 3C). Because of the patient's anxiety and restlessness, the medical team proceeded with deep sedation, endotracheal intubation, and mechanical ventilation. Telemetry recorded only four more episodes of non-sustained VT before the rhythm returned to sinus rhythm that was successfully maintained throughout the procedure. The patient was extubated after five days and was followed three months afterward; he experienced neither angina nor episodes of palpitations.

## Discussion

The term "electrical storm" (ES) was coined in the early 1990s and refers to a condition characterized by frequent occurrences of VT or VF within a brief timeframe [6]. ES is deemed to subside after a minimum of seven consecutive days without recurrent ventricular arrhythmias [2].

The pathophysiological mechanisms responsible for VA storm are not fully understood. It is probable that there is an interaction among increased sympathetic activity, abnormalities in calcium-related signalling, and a vulnerable arrhythmogenic substrate [7].

In the setting of myocardial ischemia/reperfusion, the occurrence of VA can be attributed to ischemia, no-reflow phenomenon, or reperfusion injury. While ischemic arrhythmias are typically reentrant in nature, reperfusion arrhythmias primarily operate through non-reentrant mechanisms, involving abnormal or heightened automaticity and triggered activity due to afterdepolarizations [5].

In a study conducted by Zhou et al., peri-procedure ES occurred in approximately 17.1% of patients with acute MI (AMI), and the occurrence of ES was predominantly associated with the left main artery, which was identified as the infarct-related artery, at a percentage of 55.6%, followed by right coronary artery (RCA) at a percentage of 23.7%, and LAD at a percentage of 12.4% [8].

Timely electrical defibrillation or cardioversion is the main approach used to restore hemodynamic stability in patients experiencing ES. However, excessive and frequent interventions can result in myocardial damage and may worsen arrhythmias. Hence, relying solely on electrical defibrillation or cardioversion is insufficient [1].

The management of ES, irrespective of its underlying cause and electrophysiological basis, necessitates a comprehensive approach involving multiple treatment methods, namely, device reprogramming, medication, deep sedation, and catheter ablation. There is a severe scarcity of randomized controlled data to provide guidance for therapy in the context of ES, with the majority of evidence stemming from observational studies and expert consensus [7].

Beta-receptor blockers, amiodarone, and lidocaine are the potential medications that can be utilized in this setting. Although lidocaine is the preferred pharmaceutical choice for addressing ES triggered by MI, its effectiveness diminishes when applied to cases of monomorphic VT, and it has not been proven to forestall severe VA during acute MI. Amiodarone yields excellent therapeutic outcomes for patients presenting with VT subsequent to AMI, yet its primary drawback is the QT interval prolongation. While there is a dramatic surge of cathecolamines in this instance that counteract amiodarone's action and reduce its efficacy, intravenous beta-blockers have emerged as a fundamental treatment approach to address the characteristic catecholamine surge in ES, serving as a cornerstone in antiarrhythmic drug therapy. In comparison to using amiodarone as a standalone therapy, the combination of amiodarone and beta-receptor blockers proves more effective against ES after MI [1].

In a study by Prabhu et al., 80.49% of patients who have been successfully managed were able to be discharged from the hospital after the ES, with an average hospital stay duration of 14.2 ± 2.31 days. Unfortunately, 19.5% of patients did not survive and passed away in the hospital. Among these fatalities, three deaths were linked to ES occurring in the context of ACS [2].

Our patient experienced episodes of sustained VT an hour after myocardial revascularization, preceded by ventricular ectopic beats, with a control CAG showing no evidence of stent thrombosis, which renders the reperfusion injury the most probable instance. The patient was discharged two weeks after successful rescue of ES.

## Conclusions

ES represents an arrhythmic emergency with a high likelihood of substantial morbidity and mortality. Multiple underlying mechanisms and triggers can be evoked, and post-myocardial revascularization should be ruled in depending on the context. ES due to reperfusion injury remains poorly documented and rarely reported in the literature, rendering its management challenging; as a result, a tailored therapeutic approach is crucial in the absence of specific guidelines.
